# Supplementary material for: Spatiotemporal Dynamics, Evolutionary History and Zoonotic Potential of Moroccan H9N2 Avian Influenza Viruses from 2016 to 2021
Source: Viruses. 2022 Mar 1;14(3):509. doi: 10.3390/v14030509 (PMC8951762; doi:10.3390/v14030509)
Supplement: Supplementary file 1 [file viruses-14-00509-s001.zip › Figure S6.pdf]

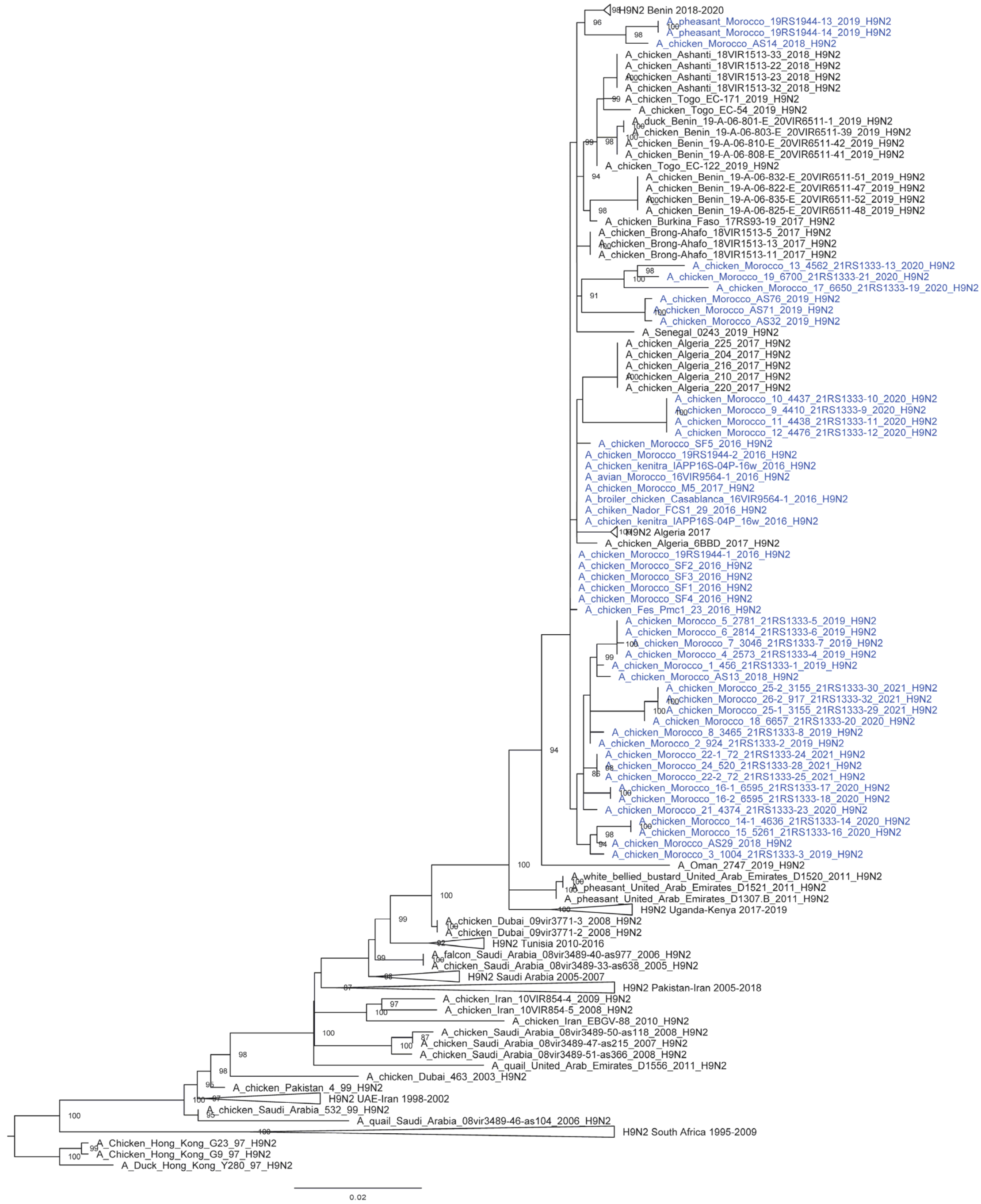

Figure S6. Maximum Likelihood phylogenetic tree of the M gene segment (IQ-TREE v.1.6.8). The H9N2 viruses from Morocco analyzed in this report are marked in blue. Ultra-fast bootstrap supports equal to or higher than 80% are indicated next to the nodes).
