# Supplementary material for: Spatiotemporal Dynamics, Evolutionary History and Zoonotic Potential of Moroccan H9N2 Avian Influenza Viruses from 2016 to 2021
Source: Viruses. 2022 Mar 1;14(3):509. doi: 10.3390/v14030509 (PMC8951762; doi:10.3390/v14030509)
Supplement: Supplementary file 1 [file viruses-14-00509-s001.zip › Table S1.pdf]

Table S1. Moroccan H9N2 viruses 2016–2021 used in the study.

Short_name	Virus	Subtype	Region	Province	Location	Date
1_BMK	A/chicken/Morocco/26-2_917_21RS1333-32/2021	H9N2	Béni Mellal-Khénifra (BMK)	Béni Mellal	Béni Mellal	2021-04-28
2_CS	A/chicken/Morocco/17_6650_21RS1333-19/2020	H9N2	Casablanca-Settat (CS)	Benslimane	Bouznika	2020-11-28
3_CS	A/broiler_chicken/Casablanca/16VIR9564-1/2016	H9N2	Casablanca-Settat (CS)	Casablanca	Casablanca	2016-02-01
4_CS	A/chicken/Morocco/14-1_4636_21RS1333-14/2020	H9N2	Casablanca-Settat (CS)	Casablanca	Casablanca	2020-03-20
5_CS	A/chicken/Morocco/24_520_21RS1333-28/2021	H9N2	Casablanca-Settat (CS)	Casablanca	Casablanca	2021-03-18
6_CS	A/pheasant/Morocco/19RS1944-13/2019	H9N2	Casablanca-Settat (CS)	Casablanca	Ain Borja	2019-03-19
7_CS	A/pheasant/Morocco/19RS1944-14/2019	H9N2	Casablanca-Settat (CS)	Casablanca	Ain Borja	2019-03-19
8_CS	A/chicken/Morocco/13_4562_21RS1333-13/2020	H9N2	Casablanca-Settat (CS)	El Jadida	El Jadida	2020-03-12
9_CS	A/chicken/Morocco/2_924_21RS1333-2/2019	H9N2	Casablanca-Settat (CS)	El Jadida	El Jadida	2019-04-03
10_FM	A/chicken/Fes/Pmc1_23/2016	H9N2	Fès-Meknès (FM)	Fes	Fes	2016-02-18
11_FM	A/chicken/Morocco/19RS1944-1/2016	H9N2	Fès-Meknès (FM)	Fes	Fes	2016-01-25
12_FM	A/chicken/Morocco/AS29/2018	H9N2	Fès-Meknès (FM)	Fes	Fes	2018-11-16
13_FM	A/chicken/Morocco/3_1004_21RS1333-3/2019	H9N2	Fès-Meknès (FM)	Fes	Fès	2019-04-11
14_FM	A/chicken/Morocco/5_2781_21RS1333-5/2019	H9N2	Fès-Meknès (FM)	Fes	Fès	2019-09-24
15_FM	A/chicken/Morocco/7_3046_21RS1333-7/2019	H9N2	Fès-Meknès (FM)	Ifrane	Ifrane	2019-10-19
16_FM	A/chicken/Morocco/AS14/2018	H9N2	Fès-Meknès (FM)	Meknes	Meknes	2018-10-24
17_FM	A/chicken/Morocco/AS76/2019	H9N2	Fès-Meknès (FM)	Meknes	Meknes	2019-02-23
18_FM	A/chicken/Morocco/AS77/2019	H9N2	Fès-Meknès (FM)	Meknes	Meknes	2019-01-17
19_FM	A/chicken/Morocco/21_4374_21RS1333-23/2020	H9N2	Fès-Meknès (FM)	Meknes	Meknès	2020-02-25
20_FM	A/chicken/Morocco/AS32/2019	H9N2	Fès-Meknès (FM)	Sefrou	Ain Chegag	2019-02-12
21_FM	A/chicken/Morocco/4_2573_21RS1333-4/2019	H9N2	Fès-Meknès (FM)	Sefrou	Sefrou	2019-09-05
22_FM	A/chicken/Morocco/10_4437_21RS1333-10/2020	H9N2	Fès-Meknès (FM)	Sefrou	Sefrou	2020-02-29
23_FM	A/chicken/Morocco/11_4438_21RS1333-11/2020	H9N2	Fès-Meknès (FM)	Sefrou	Sefrou	2020-02-29
24_CS	A/chicken/Morocco/12_4476_21RS1333-12/2020	H9N2	Casablanca-Settat (CS)	Benslimane	Bouznika	2020-03-03
25_CS	A/chicken/Morocco/8_3465_21RS1333-8/2019	H9N2	Casablanca-Settat (CS)	Benslimane	Bouznika	2019-11-29
26_FM	A/chicken/Morocco/9_4410_21RS1333-9/2020	H9N2	Fès-Meknès (FM)	EL Hajeb	EL Hajeb	2020-02-26
27_M	A/chicken/Morocco/AS13/2018	H9N2	na (M)	na	na	2018
28_M	A/chicken/Morocco/M5/2017	H9N2	na (M)	na	na	2017

29_M	A/chicken/Morocco/SF1/2016	H9N2	na (M)	na	na	2016
30_M	A/turkey/Morocco/AR166/2018	H9N2	na (M)	na	na	2018-04-01
31_RSK	A/chicken/kenitra/IAPP16S-04P_16w/2016	H9N2	Rabat-Salé-Kenitra (RSK)	Kenitra	Kenitra	2016-02-25
32_RSK	A/chicken/Morocco/19RS1944-2/2016	H9N2	Rabat-Salé-Kenitra (RSK)	Kenitra	Kenitra	2016-01-26
33_RSK	A/chicken/Morocco/25-1_3155_21RS1333-29/2021	H9N2	Rabat-Salé-Kenitra (RSK)	Kenitra	Sidi Boubker El Haj	2021-04-09
34_RSK	A/chicken/Morocco/25-2_3155_21RS1333-30/2021	H9N2	Rabat-Salé-Kenitra (RSK)	Kenitra	Sidi Boubker El Haj	2021-04-09
35_RSK	A/chicken/Morocco/AS71/2019	H9N2	Rabat-Salé-Kenitra (RSK)	Sidi Slimane	Sidi Slimane	2019-04-01
36_RSK	A/chicken/Morocco/1_456_21RS1333-1/2019	H9N2	Rabat-Salé-Kenitra (RSK)	Skhirat Temara	Skhirat Temara	2019-02-16
37_RSK	A/chicken/Morocco/18_6657_21RS1333-20/2020	H9N2	Rabat-Salé-Kenitra (RSK)	Skhirat Temara	Skhirat Temara	2020-11-30
38_RSK	A/chicken/Morocco/16-1_6595_21RS1333-17/2020	H9N2	Rabat-Salé-Kénitra (RSK)	Khemisset	Rommani	2020-11-23
39_RSK	A/chicken/Morocco/16-2_6595_21RS1333-18/2020	H9N2	Rabat-Salé-Kénitra (RSK)	Khemisset	Rommani	2020-11-23
40_R	A/chiken/Nador/FCS1_29/2016	H9N2	Rif (R)	Nador	Nador	2016-02-16
41_TTA	A/chicken/Morocco/22-1_72_21RS1333-24/2021	H9N2	Tangeri-Tetouan-Al Hoceima (TTA)	Tangeri-Assila	Tangeri	2021-02-11
