# Supplementary material for: Spatiotemporal Dynamics, Evolutionary History and Zoonotic Potential of Moroccan H9N2 Avian Influenza Viruses from 2016 to 2021
Source: Viruses. 2022 Mar 1;14(3):509. doi: 10.3390/v14030509 (PMC8951762; doi:10.3390/v14030509)
Supplement: Supplementary file 1 [file viruses-14-00509-s001.zip › Table S2.pdf]

Table S2. List of H9N2 vaccines available in Morocco.

**Commercial name of the vaccine**	**Date of authorization**	**Contenant of the vaccine**	**Targeted species**
ATLAVAC H9N2	17/12/2018	AIV H9N2 inactivated vaccine, Moroccan strain 1/16	Chicken (Broiler, layer and breeder)
ATLAVAC H9N2 + ND	23/12/2019	AIV H9N2 inactivated vaccine, Moroccan strain 1/16<br>Newcastle disease inactivated vaccine, La sota strain	Chicken (Broiler, layer and breeder)
CEVAC FLU H9 K	23/12/2019	AIV H9N2 inactivated vaccine, strain H9N2 D1991/2	Chicken (Broiler, layer and breeder)
CEVAC H9N2 MAROC	17/12/2018	AIV H9N2 inactivated vaccine, strain H9N2 H9N2-D3398.	Chicken
CEVAC NEW FLU H9 K	10/07/2020	AIV H9N2 inactivated vaccine<br>Newcastle disease inactivated vaccine, La sota strain	Chicken
PRO-VAC AIK	15/06/2017	AIV H9N2 inactivated vaccine, (A/CK/Kor/01310/2001(H9N2), CE20 strain)	Chicken (layer and breeder)
PRO-VAC AINK	02/10/2018	AIV H9N2 inactivated vaccine, (A/CK/Kor/01310/2001(H9N2), CE20 strain)<br>Newcastle disease inactivated vaccine, Ulster 2C strain	Chicken
NOBILIS INFLUENZA H9N2	09/04/2019	AIV H9N2 inactivated vaccine, A/CK/UAE/415/99 strain	Poultry (chicken and turkey)
NOBILIS INFLUENZA H9N2+ND	15/06/2017	AIV H9N2 inactivated vaccine, AG415 strain<br>Newcastle disease inactivated vaccine, Clone30 strain	Chicken
GALLIMUNE 208 ND+FLU H9 M.E.	02/10/2018	AIV H9N2 inactivated vaccine<br>Newcastle disease inactivated vaccine, Ulster 2C strain	Chicken (Broiler, layer and breeder)
GALLIMUNE FLU H9 M.E.	02/10/2018	AIV H9N2 inactivated vaccine	Chicken (Broiler, layer and breeder)
ME FLUVAC H9 0,3%	05/01/2018	AIV H9N2 inactivated vaccine, Chicken/Egypte/114940V/ NLQP/2011starin	Poultry (Chicken, duck and goose).
IZOVAC AVIFLU		AIV H9N2 inactivated vaccine, (A/Turkey/Italy/245/84 (H9N2) strain	Chicken (Broiler, layer and breeder)
