# Supplementary figures and images for: Spatiotemporal Dynamics, Evolutionary History and Zoonotic Potential of Moroccan H9N2 Avian Influenza Viruses from 2016 to 2021
Source: Viruses. 2022 Mar 1;14(3):509. doi: 10.3390/v14030509 (PMC8951762; doi:10.3390/v14030509)

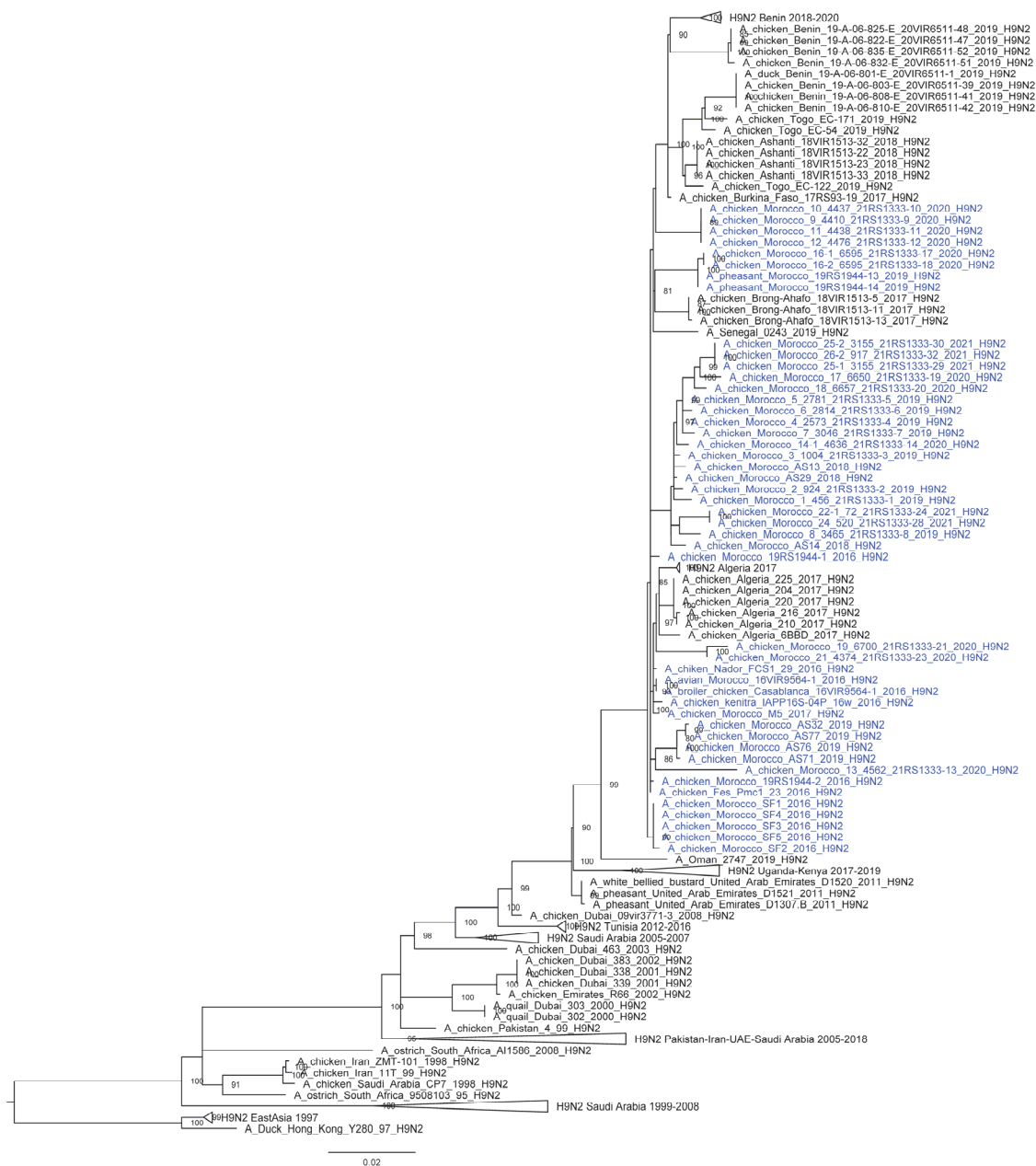

Supplement: Supplementary file 1 [file viruses-14-00509-s001.zip › Figure S5.pdf]

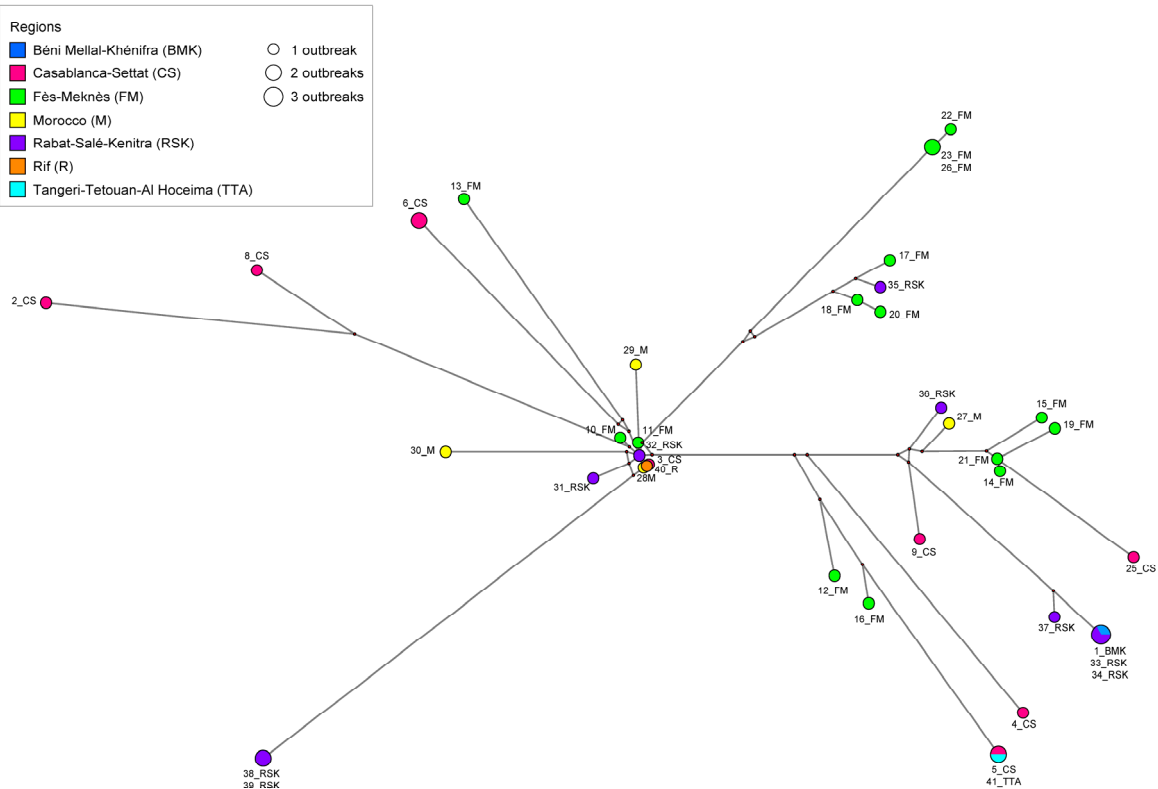

**Figure S8.** the HA median-joining network for Moroccan regions.

Supplement: Supplementary file 1 [file viruses-14-00509-s001.zip › Figure S8.pdf]
